# Plasticity facilitates sustainable growth in the commons

**DOI:** 10.1098/rsif.2012.1006

**Published:** 2013-04-06

**Authors:** Matteo Cavaliere, Juan F. Poyatos

**Affiliations:** Logic of Genomic Systems Laboratory (CNB-CSIC), Madrid, Spain

**Keywords:** sustainability, tragedy of commons, bounded rationality, microbial cooperation, ecological rationality

## Abstract

In the commons, communities whose growth depends on public good, individuals often rely on surprisingly simple strategies, or heuristics, to decide whether to contribute to the shared resource (at risk of exploitation by free-riders). Although this appears a limitation, we show here how four heuristics lead to sustainable growth when coupled to specific ecological constraints. The two simplest ones—contribute permanently or switch stochastically between contributing or not—are first shown to bring sustainability when the public good efficiently promotes growth. If efficiency declines and the commons is structured in small groups, the most effective strategy resides in contributing only when a majority of individuals are also contributors. In contrast, when group size becomes large, the most effective behaviour follows a minimal-effort rule: contribute only when it is strictly necessary. Both plastic strategies are observed in natural scenarios across scales that present them as relevant social motifs for the sustainable management of public goods.

## 1. Introduction

In many biological, social and economic systems, there exists a continuous interplay between individual actions and collective dynamics [1–3]. This interplay becomes particularly significant when the individual decisions on how to contribute to a public resource ultimately determine the sustainability of the whole. The choice of contributing—that implies personal costs—favours not only community growth but also promotes the appearance of *free-riders*. These agents take advantage of the public good (PG), spread in the population and can eventually bring its collapse [4].

This predicted scenario does not correspond however with many observations. Stable communities whose growth is based on PG are indeed observed at all scales; from microbial aggregates, e.g. biofilms depending on the individual contribution of extracellular substances, e.g. [5,6], to human commons, e.g. fisheries, forests, etc. (note that in some of these cases the choice is not so much to contribute but to make appropriate use of a shared resource) [7,8]. These findings triggered the interest in understanding the type of behavioural strategies that could be adopted by individuals to help avoid collapse and how such outcomes could further depend on specific structural features characterizing the community.

Notably, the adoption of simple strategies appears to be efficient enough to promote sustainability [9]. Simple rules contrast the idea of elaborated behaviours that allow individuals to optimally maximize benefits, a null model particularly extended in studies of human commons. In this context, the relevance of elementary strategies (or heuristics) was first investigated by Herbert Simon who also pioneered the essential connection between heuristics and the particular environment where they are to be applied [10] (see also [11–13]). In a broader perspective, heuristics—sometimes interpreted as behavioural ‘limitations’—can then represent effective strategies to deal with complex ecological constraints—a consideration that applies to bacterial, animal and human decision-making circumstances [7,9,14–16].

**Figure 1.**
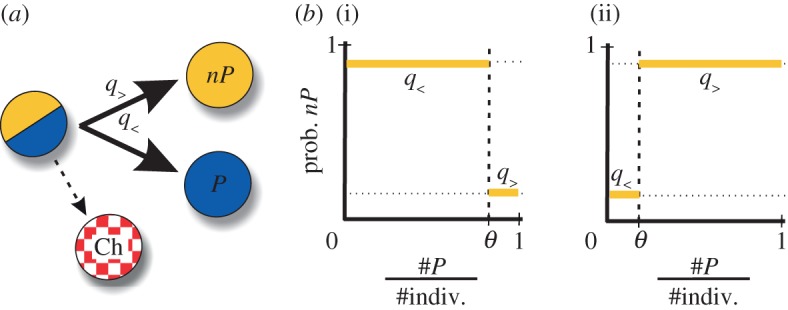
Individuals as plastic producers. (*a*) A plastic producer can contribute (producer state, *P*) or not (non-producer state, *nP*) to the PG. This choice is made after each interaction and is conditioned to the specific group composition experienced, i.e. the ratio between the number of *P*s and the total number of individuals in the group. If this ratio is bigger, or equal, than a particular threshold value *θ*, an agent becomes *nP* with probability *q*_>_ (if the ratio is smaller, it becomes *nP* with *q*_<_). Cheaters (Ch), i.e. agents that are permanently in the *nP* state, arise from plastic producers by mutation. (*b*) Following this, a constitutive producer corresponds to the case *q*_>_=*q*_<_=0, while a stochastic producer exhibits non-zero *q*_>_ = *q*_<_. *Positive* plastic producers (i) are characterized by a relatively large *θ*. This implies that they hardly become *nP* if the group they experienced was mostly constituted by *P*s (as they are also defined by a small *q*_>_); if not, they express *nP* with high probability (large *q*_<_). In contrast, *negative* plastic producers (ii) present a fairly small *θ*. This means that they express *nP* with high probability (as they are also defined by a large *q*_>_) unless they were part of groups with few *P*s, in which case they hardly express *nP* (small *q*_<_). Note that the number of individuals (#indiv.) can be less or equal than the group size (*N*).

In this manuscript, we examine how simple strategies, which exhibit limited information processing (figure 1*a*), can nevertheless be adjusted to exploit certain environmental constraints to attain sustainable growth. We modify these constraints by changing core structural factors characterizing the community, such as the size of its constituent groups (*N*) or the resource characteristics, i.e. PG efficiency (*r*). To this aim, we model the commons by means of a stylized ecological PG model in which a finite population of agents is organized in groups where they are involved in a PG game, and the supply of PG determines population density (a model originally introduced in [17] for infinitely large populations).

We first find that permanent production of PG works when its creation efficiently induces growth and the commons is structured in relatively small groups. We then observe that a simple strategy that stochastically alternates between contribution and non-contribution enlarges the range of commons where its adoption leads to sustainability (when compared with the previous case). Finally, we discover two opposite plastic heuristics—in which a simple sensing mechanism is at work (figure 1*b*)—to be effective in two contrasting environmental situations. While *positive* plasticity (contribute only when most individuals in the previous interaction group were contributing) works for low efficiency and small groups, *negative* plasticity (contribute merely when it is strictly necessary, i.e. individuals in the past group hardly contributed) does it for high efficiency and large groups.

## 2. Results

### 2.1. Constitutive production and the risks of the commons

**Figure 2.**
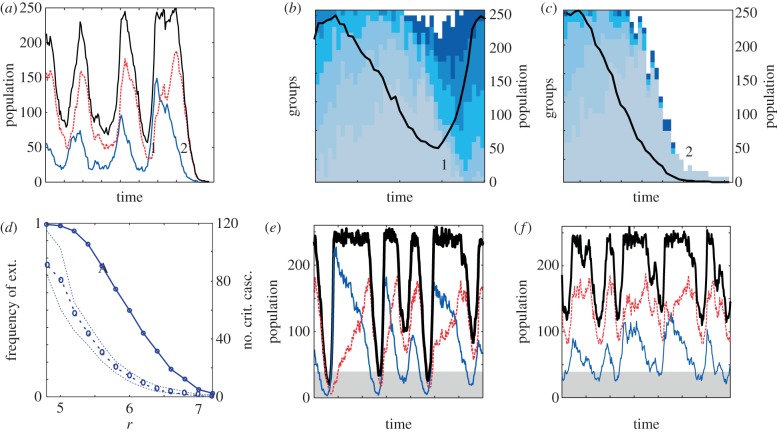
Growth based on constitutive producers is not sustainable for weakly efficient public good. (*a*) Recovery and extinction in a population composed by constitutive *P*s and cheaters (colour code as in figure 1; total population in black). (*b*,*c*) Each group in the population (squares) is coloured in different blue tones in a recovery (*b*) (around 1 in (*a*)) or an extinction (*c*) (around 2 in (*a*)) event. Group composition ranges from all *P* (dark blue) to all cheaters (light blue); white denotes empty groups. Note the enrichment of groups with only *P* immediately after each population decay. Unsuccessful replication of these initial groups causes population collapse. (*d*) Frequency of extinctions (solid line) and median number of critical cascades (dashed line) as a function of *r*. (*e*,*f*) Characteristic trace of regimes with low (*e*) and high (*f*) *r*. The population crosses more often the critical density region (highlighted in grey) at low *r* for an equivalent time window. A critical cascade is observed when the population crosses the critical density threshold, fixed to 30 (i.e. = *k*/10). Each point in (*d*) is the median (and 25/75% percentiles) of the average number of critical cascades obtained by considering all simulations that did not go extinct in 1000 independent runs of 6 × 10^5^ steps. Parameters: *k* = 300, *N* = 10, *ν* = 5 × 10^−6^, *δ* = 0.2, *c* = 1 (all panels); (*a*,*b*,*c*) *r* = 4, (*e*) *r* = 4.6 and (*f*) *r* = 6.5.

We first examined the consequences of the simplest possible strategy: permanent and indiscriminate production of PG. This strategy maximizes growth but additionally favours the emergence of cheaters (that arise by mutation from an all*P* population). Cheaters rapidly invade a resident population but also cause its decline, because of the coupled decay in PG (less *P*s) that limits growth (figure 2*a*).

The population cascade associated with cheater expansion can unexpectedly lead to its recovery. This is linked to the group structure of the interactions in the commons. Sufficiently small density causes the appearance of groups primarily composed of only *P*s or only cheaters (figure 2*a*,*b*) that multiplies the replication of constitutive *P* and reduces that of cheaters, both processes contributing to the recovery of the population (when enough inter-group composition variance is generated, in what is known as the Simpson's paradox, an effect associated with the metapopulation dynamics [17,18], see also the electronic supplementary material).

However, this recovery dynamics includes an added risk, since the low density could precipitate population extinction by stochastic demographic effects [19], figure 2*a*,*c*. Risk is raised when the population repeatedly exhibits *critical cascades*, i.e. declines in density below a particular minimal value. The final outcome between recovery and extinction is strongly determined by the intrinsic properties of the commons. Constitutive production reveals in this way as a successful strategy when the PG efficiently determines growth (*r* sufficiently high, figure 2*d*–*f*; the influence of *r* was also studied in the deterministic model [17]) or when groups within the commons are relatively small (controlling for *r*, electronic supplementary material, figure S2).

### 2.2. Stochastic production can reduce the risks

We analysed a second strategy in which individuals choose randomly whether to contribute or not to the PG (i.e. they can sometimes decide to free ride). Specifically, agents present a *nP* state with probability *q*_>_ (or, conversely, an *P* state with 1−*q*_>_; figure 1). Therefore, stochastic producers are totally unable to sense the composition of their interaction group (the amount of available PG).

A homogeneous population of stochastic producers generates a constant sub-population of *nP*s that decreases PG levels with two consequences. It can reduce not only the chances of cheaters to replicate (which favours sustainability), but also drive the system to extinction even without any cheater present—by causing severe PG reduction. We quantified this trade-off by computing the number of critical cascades, as before, and their duration (i.e. number of consecutive steps below the minimal density threshold). Increasing *q*_>_ reduces the number of critical cascades but steadily increases cascade duration (that reflects the delay in the appearance of all *P* groups associated with population recovery).

**Figure 3.**
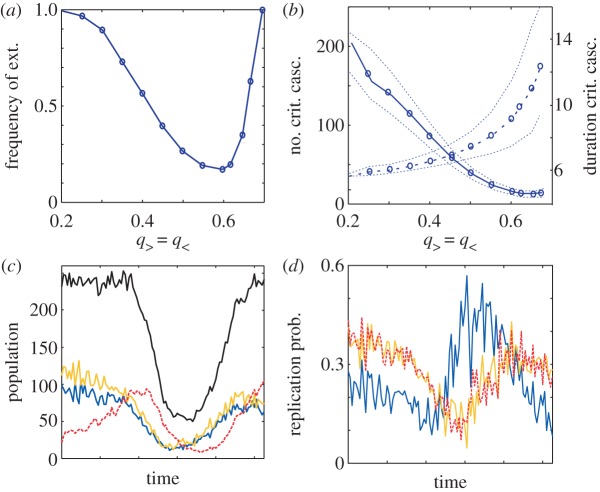
Stochastic producers can reduce extinctions. (*a*) Frequency of extinctions as a function of the probability *q*_>_ to stochastically express a *nP* state (*q*_>_= *q*_<_, see figure 1). There exists an optimal *q*_>_ that reduces extinctions by decreasing the number of critical cascades (solid line in (*b*)) trading-off for their duration (dashed line in (*b*)). (*c*,*d*) Characteristic dynamics of a stochastic producer with optimal switching rate (*q*_>_=0.55, colour code as in figure 1, black curve denotes total population). Note the limited replication rate of cheaters (generally below approx. 50%) even when population is high. Cheater advantage is stronger when agents constitutively produce PG (electronic supplementary material, figure S3). A critical cascade is observed when the population crosses the critical density threshold, fixed to 30 (i.e. = *k*/10), while the duration of a critical cascade is the average number of consecutive steps the population stays below the critical density threshold. Each point in (*b*) is the median (and 25/75% percentiles) obtained by considering all simulations that do not go extinct in 1000 independent runs with 6 × 10^5^ steps. Parameters: *k* = 300, *N* = 10, *r* = 4, *c* = 1, *δ* = 0.2.

This trade-off indicates an optimal *q*_>_ that minimizes the frequency of extinctions and defines the exact stochastic rate for a successful strategy (figure 3*a*,*b*). When agents follow this optimal stochastic heuristic, cheater replication is limited even for high densities (generally below 50%, figure 3*c*,*d*), population oscillations are damped and extinction risks reduced. This dynamics contrasts with the constitutive heuristics scenario in which cheater replication can reach very high values in dense populations (see the electronic supplementary material, figure S3).

Sustainability is thus attained in a wider range of commons (in terms of *r* and *N*) when agents followed a stochastic strategy. However, this range is still limited. A relative decrease in *r* (electronic supplementary material, figure S4) or an increase in *N* (see the electronic supplementary material, figure S5) once more implies an increment in the number of critical cascades and in this way of extinctions. Alternative strategies are required in those commons.

### 2.3. Plastic cooperation favours sustainable growth

To analyse whether the addition of some basic information-processing features could direct to more effective heuristics (in commons where growth is unsustainable with the use of constitutive or stochastic production), we examined a third strategy that includes a simple sensing mechanism. This sensing permits agents to evaluate the relative abundance of *P*s in the group where they most recently played the game (this can be estimated by means of the amount of PG received).

If the PG obtained in the previous interaction is above (below) a particular threshold *θ*, individuals exhibit the *nP* phenotype with probability *q*_>_ (*q*_<_). This defines a general plastic producer (figure 1*a*). We then studied the dynamics of a population of individuals exhibiting different plastic heuristics (distinct *q*_>_, *q*_<_, *θ*) in a range of commons (characterized by *r* and *N*). By using an exhaustive analysis (of all possible strategies and commons conditions, see electronic supplementary material), we were able to identify two specific plastic heuristics that lead to sustainable growth in a wider range of commons.

#### 2.3.1. Positive plasticity is the most effective strategy for small groups and low efficiency

**Figure 4.**
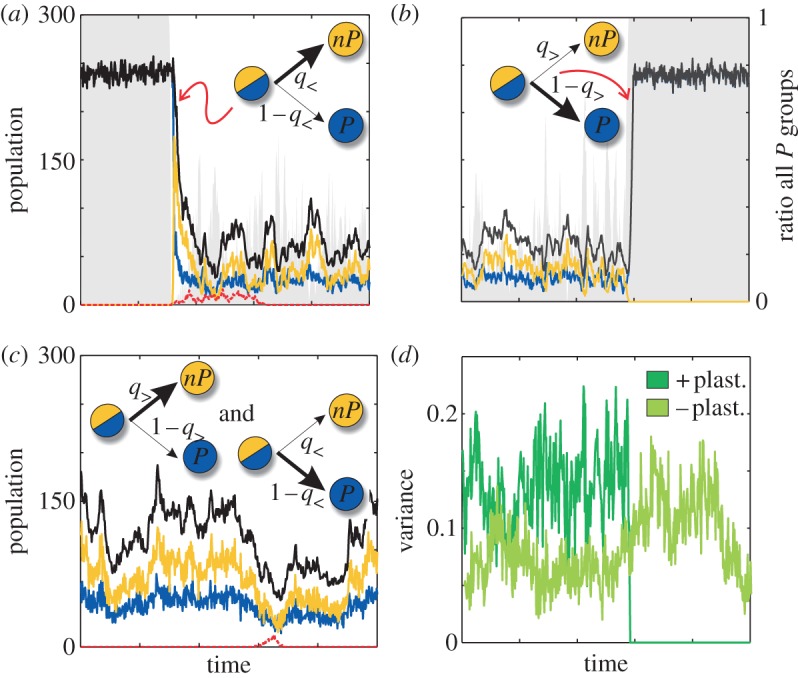
Positive and negative plastic producers include a sensing mechanism. (*a*) Positive plastic producers expressing a *P* state quickly switch to *nP* as response to cheaters. This causes a fast reduction of *P*s overall (arrow thickness in inset cartoons denotes preferred individual decisions). After cheater invasion is stopped, the population exhibits coexistence of *P* and *nP* to finally evolve to an all *P* scenario (*b*) (for better visualization we show only a limited part of the coexistence). Shading areas in (*a*,*b*) denote the relative amount of groups composed by only *P*s. (*c*) A population of negative plastic producers is characterized by its permanent low density favoured by the constant presence of *nP* which helps controlling cheaters invasion. (*d*) Positive plasticity transiently modifies the inter-group variance to control cheaters and stop the emergence of *nP*s. This contrasts with the relatively constant variance observed in a population of negative plastic producers (variances correspond to time series (*b*) and (*c*), respectively). Plastic producer definitions and colour code as figure 1, with *q*_>_=0, *q*_<_=0.7 and *θ* = 1 for positive plasticity and *q*_>_ = 0.7, *q*_<_ = 0 and *θ* = 0.1 for negative; black curve describes total population. Parameters: *k* = 300, *N* = 10, *δ* = 0.2, *c* = 1, *ν* = 5 × 10^−6^ (all panels); (*a*,*b*) *r* = 2 and (*c*) *r* = 2.2.

The most valuable heuristic in commons characterized by small groups and low PG efficiency *r* consists of contributing to PG only if most members of the agent's recent interaction group were also contributing. Individuals that follow this heuristic (positive plastic producers) immediately react to the presence of *nP* (or cheaters) in their past interaction, becoming *nP* themselves (formally, they present a small *q*_>_, but a large *q*_<_ and *θ*, figure 1*b*). The appearance of cheaters in a population of positive plastic producers (in state *P*) consequently originates an immediate decrease of PG in each group which triggers the remaining *P*s to stop contributing and switch to *nP* (figure 4*a*).

As the population declines, the number of groups exclusively formed by the residual plastic individuals in the *P* state increase (shading in figure 4*a*,*b*). This situation drives the system to the ‘recovery regime’ where inter-group variance makes the Simpson's paradox decisive once more (figure 4*d*). Note that this recovery dynamics, characteristic of the structure of commons, is enhanced by the heuristic at work: *P* individuals that experienced groups of only *P*s keep contributing with high probability. The whole process stops the creation of *nP*s, expels cheaters and takes the population back to an all*P* regime, an absorbing state of the system (figure 4*b*). Once the population is uniquely constituted by *P*s, it remains in this homogeneous state until new cheaters arise.

If groups are relatively large, the mechanisms just described fail. While the reaction to cheater invasion is similar, the enrichment of all*P* groups is delayed. Only when the population level becomes very low, these groups start to arise but, as we discussed earlier, this regime increases the chance of demographic extinctions. These collapses are not totally avoided in commons displaying higher *r*. In these cases, large group size *N* and large efficiency *r*, we identified an alternative plastic heuristic that can assist sustainable growth.

#### 2.3.2. Negative plasticity is the most effective strategy for large groups and high efficiency

Individuals following a minimal-effort (plastic) heuristic are the ones that most strongly bring sustainability in commons structured in large groups, and where the supply of PG efficiently determines growth. These negative plastic producers present a *nP* state unless the amount of PG in their latter interaction group is below a minimal threshold that could in the end impede growth (formally, they exhibit a large *q*_>_, but small *q*_<_ and *θ*, see figure 1*b*). Thus, a population constituted by negative plastic producers is constantly at low density, independently of the presence of cheaters.

The low-density regime is maintained as a dynamical equilibrium in which an excess of *P*s makes individuals to become *nP*, since many *P*s are observed in each group, while the successive lack of PG (and of *P*s in the groups) is compensated by showing again the *P* state. In this scenario, the emergence of cheaters by mutation is indirectly controlled by the high abundance of *nP* already in the resident population, which in turn reduces cheater presence and chance of invasion (figure 4*c*).

As negative plasticity strongly relies on the abundance (by default) of *nP* agents, this heuristic requires that the PG produced by those that contribute must transform very efficiently into growth. For this reason, negative plasticity is successful only when *r* is above a certain minimal value. The abundant presence of *nP* agents implies in this way that the resistance to cheaters is obtained by paying the price of a limited growth. When compared with a population of positive plastic producers, the mechanisms of recovery do not rely so much on the temporal increase of inter-group variance (that brings the ‘recovery regime’) but on the presence of a relatively constant and adequate inter-group diversity (figure 4*d*).

## 3. Discussion

Communities whose growth depends on a PG contributed by their members present a fundamental instability associated with the emergence of free-riders (cheaters) that do not contribute but use the accessible PG. This instability—at its core a problem of maintenance of cooperation—produces direct ecological consequences, i.e. the collapse of the population.

This ecological scenario immediately defines a characteristic ‘environment’ in which individuals following simple strategies are to ‘solve’ a precise task: to attain the sustainable growth of the collective. We analysed this situation by considering limitations upon the decision-making capacities (figure 1) and also modifications of the specific attributes of the environment (*r* and *N*) where decisions are taken [10]. Our work then links bounded rationality [10], heuristics on public-good settings [7] and ecological rationality [12].

The analysis of the simplest heuristic, constitutive production of PG, reveals the core ecological dynamics (figure 2*a*, see also [17] for an analysis of this dynamics in infinite populations). By avoiding production costs, cheaters can spread in a population of (constitutive) *P*s consequently reducing population density owing to PG depletion. The resultant low densities induce the formation of between-group differences (groups dominantly constituted by *P*s or cheaters, figure 2*b*,*c*). This high inter-group variance causes individuals in groups dominantly composed by *P*s to receive larger payoffs, i.e. present higher replication rates. Differential growth leads to population recovery, as *P* agents are the ones strongly contributing to the next generations, figure 2*a*,*b*, an application of the Simpson's paradox to multilevel selection, [18]. Low densities help then to promote *P*s (i.e. cooperation) in such structured populations.

Low densities originate a complementary ecological effect, when populations undergo demographic extinctions [19] instead of recovery. We captured these processes by quantifying the number of critical cascades—the number of times that the population is below a minimal density threshold. A decrease in *r* or an increase of *N* increases the number of critical cascades and the frequency of extinctions if PG is constitutively generated (figure 2*d* and electronic supplementary material, figure S2). Hence, permanent production of PG does not always drive sustainable growth.

We identified two additional heuristics. The simplest one, in which no external information is processed, consists of switching stochastically between contribution and non-contribution, i.e. individuals decide randomly to free-ride. This behaviour (defined by an opportune optimal switching rate, figure 3*a*,*b*) is generally more effective than constitutive production, but fails again when *r* becomes smaller or *N* larger (see the electronic supplementary material, figures S4 and S5).

The third heuristic is based on conditional contribution. This is implemented by means of a simple sensing mechanism that allows individuals to estimate the composition of their recent interaction group and alter their behaviour accordingly. Modifying the two key structural attributes of the commons lets us identify two contrasting conditional strategies.

**Figure 5.**
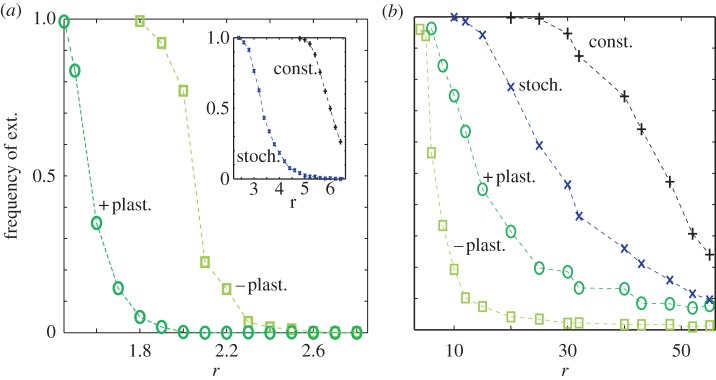
Individual strategies differentially manage sustainability in the commons. Extinction frequencies observed by individuals exhibiting constitutive (black), stochastic (blue) and positive/negative (dark/light green) plasticity in a population structured in small ((*a*), *N* = 10) or large ((*b*), *N* = 60) groups (as a function of *r*). We considered 1000 independent runs with 6 × 10^5^ steps (electronic supplementary material, figures S8 and S9 considered a different *k* and longer time series, respectively). For the curves corresponding to positive, negative plasticity and stochastic producers, the values plotted correspond to the minimal extinction frequency obtained by considering all possible instances of positive, negative and stochastic producers, respectively (see electronic supplementary material, figures S5–S7 for details). Other parameters are *k* = 300, *c* = 1, *δ* = 0.2, *ν* = 5 × 10^−6^.

For low *r* (and sufficiently small group size *N*) positive plasticity is the most advantageous strategy (figure 5*a*). This is related to its highly reactive response to cheaters. In fact, positive plastic producers stop producing PG when few cheaters (or few *nP*s) are detected in their earlier interaction group. This response immediately directs to minimal densities (figure 4*a*) and a successive strong recovery to the population carrying capacity (figure 4*b*). Interestingly, the reaction to cheaters invasions (consisting in the rapid increase of *nP*s) is promptly interrupted as a result of the feedback between the threshold-like decision and group assortment of *P*s (created by the combination of low density and small group size, figure 4*a*). This example emphasizes how heuristics associated with limited information processing (the limitation corresponds in this case to the inability of individuals to distinguish the presence of cheaters from that of plastic producers in the *nP* state) are still efficient owing to the specific ecological structure where they are applied [10–13].

Positive plasticity does not work when the commons is structured in large groups. In this case, negative plasticity emerges comparatively as a better strategy (figure 5*b*). This minimal effort behaviour [20] maintains the population in a dynamical equilibrium with the largest possible frequency of *nP*s that minimizes cheaters advantage but is compatible with population growth. Negative plasticity is in this sense an advanced version of stochastic production with the individual ability to switch back to a *P* state when population density reaches critical values. Either strategy could become unnecessarily detrimental for growth in the absence of cheaters.

Thus, the use of different decision-making strategies clearly causes divergent sustainability outcomes when controlling for community structure (i.e, when both *N* and *r* is fixed, figure 5). One could further ask whether these strategies are observed in natural scenarios (characterized by a PG dilemma). We suggest that this is the case. One of these scenarios corresponds to the ample use of PGs by microbes that include extracellular enzymes, antibiotics, siderophores and quorum-sensing molecules [21,22]. In this context, phenotypic noise, similar to the stochastic production strategy, is present as a broad form of bet hedging [23] or in the stochastic expression of virulence factors, e.g. [24]. Moreover, in many instances, production of PGs is activated/terminated at high cell densities [25]; expression of bacteriocins is reduced when the population density is low by a quorum-sensing system [26], as in the discussed positive plasticity. On the other hand, negative plasticity resembles the notion of facultative cheating [27], a cellular strategy implemented by different molecular mechanisms: generation of iron-scavenging pyoverdin molecules—iron being an essential PG in some environments—is reduced when enough molecules are already in the environment minimizing in this way the ability of cheaters to invade [28]; production of invertase, a PG necessary to hydrolyse glucose, is also repressed when not needed [27]. Constitutive and plastic strategies are now also being studied with synthetic bacterial communities [29,30].

At a very different scale, the proposed ecological model suggests a link between individual heuristics and plastic cooperation in human commons. Recent studies have discussed the use of heuristics in PG games [31], together with various forms of plastic cooperation with a variable degree of individual investment linked to group size [9,32–34] and to the amount of contributing individuals in the group [35,36]. Interestingly, experiments with PG games discovered the presence of plastic strategies [36] similar to those found to be successful for the sustainability of the commons: conditional cooperation where individuals contribute if others do so, resembling our positive plasticity, and *hump-shaped* cooperators that contribute only up to a maximum, resembling the presented negative plasticity.

That all the situations above correspond to very separate scenarios indicates that these heuristics could be fundamental building blocks in the assembly of this type of social arenas and, more broadly, in the maintenance of cooperation in structured populations. Overall, these findings stress that beyond the importance of structural factors, like PG efficiency and group structure, the sustainability of the commons should be understood as the appropriate integration of ecological dynamics and individual information-processing abilities.

## 4. Methods

### 4.1. Public good games

PG games are used to model social dilemmas in which the optimal behaviour of the individual conflicts with the best outcome of the collective [3]. The simplest of these models is the one-shot PG game [37] in which agents can contribute (cooperators) or not (defectors) to the PG in groups of size *N*. Contributing implies a cost *c* to the agents. Group contributions are then summed, multiplied by a reward factor *r* (that determines the efficiency of the investments and the attractiveness of the PG) and redistributed to all group members, *irrespectively* of their contribution.

This implies that in a group (of size *N*) with *i* cooperators, defectors would receive *icr/N* as payoff while cooperators would obtain *icr/N*−*c*. It is always better to defect than to cooperate, regardless of the number of cooperators in the group, since defection is associated with a higher payoff. In the framework of evolutionary game theory where payoff is equated to fitness, defectors reproduce faster and outcompete cooperators. This would lead ultimately to a limiting scenario of a population with only defectors and zero payoffs, i.e. population collapse. Cooperators will only be maintained if specific mechanisms that enforce their assortment are present. One of these mechanisms considers a population of cooperators and defectors structured in randomly formed groups in which PG interactions take place (Hamilton's group selection model [38]).

### 4.2. Computational model

We used a computational model based on Hamilton's group selection model that incorporates ecological dynamics and individual decision-making (individuals are able to choose dynamically their future phenotype, i.e. cooperator or defector, according to their previous experience). The associated PG game is characterized by the parameters *N*, *r* and *c* (group size, efficiency and cost of the PG, respectively, where we fixed*c* = 1 without loss of generality). Since cooperation/defection involves the production/non-production of a PG we termed cooperators as producers (*P*) and defectors as non-producers (*nP*); cheaters express constitutively the *nP* state, see figure 1*a*.

Every simulation starts with an initial population constituted by a common pool of *k* identical plastic agents in the *P* state, where *k* is the maximal population size (carrying capacity), to be updated in a *sequential* way as follows: (i) the common pool is divided in randomly formed groups of size *N* (i.e. *N* is the total number of individuals and empty spaces in each group). The number of formed groups is then ⌊*k/N*⌋. (ii) In each one of the (non-empty) groups, a one-shot PG game is played. This means that agents in the *nP* state and cheaters receive the payoff P_*nP*_ = *icr*/(*i* + *j* + *w*), while agents in the *P* state receive the same payoff minus a cost, i.e. P_*P*_ = P_*nP*_ − *c*; with *i,j* and *w* being the number of *P*s, *nP*s and cheaters in the group, respectively, and *i* + *j* + *w* ≤ *N*. After the interaction the grouping of individuals is dissolved. (iii) Each plastic agent adjusts its state according to the relative abundance of *P*s that experienced in the group where it played the game and the triplet (*q*_>_, *q*_<_, *θ*) as described in figure 1*a*. (iv) Each individual can replicate (duplicate) with a probability that is calculated by dividing its payoff by the maximal possible one (i.e. the payoff obtained by a *nP*, or equivalently a cheater, in a group of *N*−1 *P*s). Each individual that replicates generates an offspring that is either a cheater (with probability *ν*) or an identical offspring (with probability 1−*ν*). The current state of the parent is used as initial state of the offspring. (v) Individuals are removed with probability *δ* (individual death rate).

In simpler words, the life cycle of the computational model is characterized by two distinct *stages*. In stage I (steps i–ii), the population is structured in evenly sized randomly formed groups in which the PG game is played. In stage II (steps iii–v, after groups disappear), each individual chooses its successive phenotype and then replicates according to the group composition (and payoff) experienced in stage I. Replication can happen only when the current total population is less than the maximal population size, *k*, i.e. there exits empty space (empty spaces are calculated by considering *k* minus the current amount of individuals in the population). If more individuals could replicate than the available empty space, only a random subset of them ultimately replicates (of size the number of empty spaces available). A cartoon describing the life cycle of the computational model is presented in the electronic supplementary material, figure S1.
